# Pan-cancer Analysis Combined with Experiments Deciphers PHB Regulation for Breast Cancer Cell Survival and Predicts Biomarker Function

**DOI:** 10.2174/0113862073266248231024113533

**Published:** 2024-11-07

**Authors:** Xiaoyan Zhou, Yue Li, Jiali Liu, Wei Lu, Sanyuan Liu, Jing Li, Qian He

**Affiliations:** 1 Department of Clinical Laboratories, Second Affiliated Hospital of Xi’an Jiaotong University, Xi’an Jiaotong University, Xi’an, China

**Keywords:** Breast cancer, prohibitin, bioinformatics, proliferation, invasion, pan-cancer analysis, TCGA

## Abstract

**Background:**

Breast carcinoma has become the leading fatal disease among women. The location of *prohibitin* in the chromosome is close to the breast cancer susceptibility gene 1 (BRCA1). Accumulated research reported that *prohibitin* could interact with a variety of transcription factors and cell cycle-regulating proteins.

**Objective:**

This present study aims to comprehensively explore and reveal the biological functions of prohibitin on breast cancer *via* The Cancer Genome Atlas (TCGA) and validation experiment *in vitro*.

**Methods:**

Exploring the expression level of prohibitin across 27 tumors based on the TGGA database by bioinformatic methods and its relationship with tumor immune infiltration. Furthermore, we thus analyzed the biological roles of prohibitin on human breast cancer cell line MCF-7 with pEGFP-prohibitin overexpression plasmid by western blotting and transwell-assay.

**Results:**

Firstly, we found prohibitin is overexpressed in most tumors based on The Cancer Genome Atlas database, and the negative relationships between *prohibitin* and tumors infiltrating lymphocytes including B lymphocyte, CD4 T lymphocyte, CD8 T lymphocyte, Neutrophil, Macrophage and Dendritic, and its significant correlation with the prognosis of human cancer. *In vitro,* expression not only inhibited cell viability and invasive abilities but also increased the apoptosis percentage of cells with a decreased percentage of the S phase and an increased G2 phase. The reduction of Bcl-2 was observed when prohibitin was upregulated, although the expression of E2F-1 did not change.

**Conclusion:**

Although prohibitin is over-expressed in various cancer types, it functions as an important tumor suppressor that may suppress breast cancer cell proliferation and the invasive ability of MCF-7 by influencing its DNA synthesis and promoting cell apoptosis. All these may be likely associated with P53, erbB-2, and Bcl-2.

## 1 INTRODUCTION

Breast carcinoma has become the leading fatal disease and health problem among women [1-7]. Although many measures and scientific technologies have been applied to screening, diagnosis, therapy, and survival for breast cancer [8-11], they remain unavailable and insufficient for women due to the financial burden over the world [2, 11-13]. Breast cancer has a well-known complicated etiology and genetic characteristics [7, 14], which leads to classification diversity, clinical manifestations complexity, therapeutic disparities, and therapeutic specificity [15]. The morbidity and mortality rates were different among racial and ethnic groups [3]. For example, white women had the highest breast cancer incidence rates among women aged 40 years and older, but African American women gently increased with the poorest breast cancer survival of any racial/ethnic group [2] from 2006 to 2010. Lacking awareness of early detection, effective individualized therapy, or adequate financial support, the control of breast cancer is not still satisfied [16-18]. Fortunately, with timely screening and intervention of early breast cancer [6, 20], clinical use of tumor biomarkers [19-21], and continuous study of molecular mechanisms [22], the mortality rate of breast cancer has decreased gently [6]. Personalized treatment has gradually penetrated the consciousness of clinical researchers, which has brought good news to the survival rate and quality of life for breast cancer patients [3, 6, 23], especially in elderly patients. Various guidelines also suggest that precision treatment and improving quality of life should be given priority [6, 22]. In China, morbidity rates of breast cancer were becoming younger year by year [24] which same in other region of the world such as America [25]. Young women are more likely to develop more aggressive subtypes of breast cancer and have a unique biological feature, its survival rates in young women remain lower than those in older women which refers to more likely psychosocial concerns [25-28]. Therefore, it is urgently necessary to improve the diagnosis and treatments for different age stages, Complex molecular mechanisms, and highly specific biomarkers are eagerly called for individualized treatment of breast cancer. Bioinformatics analysis of high-throughput multiomics data including TCGA would be helpful to comprehensively identify the characteristics and roles of tumor-related genes, to explore their important biological roles in the progression of breast cancer [29].

Prohibitin (PHB/phb) is a highly conserved nuclear matrix protein with multiple functions, including proliferation, apoptosis, transcription, posttranslational modification, signal transduction, and energy metabolism [30-32], and it consists of two subtypes phb1 and phb2 in the mitochondria which acting as a molecular chaperone to maintain mitochondrial homeostasis [31, 32]. It is found to be associated with breast cancer [33, 34] in addition to liver cancer [35, 36], lung cancer, cervical cancer, and ovarian cancer [37] extensively. PHB is located extensively in the cell, such as the cytomembrane, cell unclear and mitochondria inner membrane, *etc*., and the location decides its function [30]. What’s more, PHB can shuttle between the nucleus and organelles under several stimulates [38, 39]. Thus, PHB may play diverse functions depending on transport. In general, nucleus PHB acts as a transcriptional regulation factor, membrane PHB plays signal transduction, and cytoplasm PHB participates in metabolism to keep a steady state [30]. Accumulated research reported PHB could participate in kinds of signal paths and interact with a variety of transcription factors and cell cycle regulating proteins [32, 40, 41]. However, the explicit mechanism has not been illuminated. The location of PHB in the chromosome (17q21) is close to breast cancer susceptibility gene 1 (BRCA1) which links it to breast cancer easily [35]. There is evidence indicating the mutations of BRCA1 were the key issue for breast cancer through bioinformatic methods [29, 42]. Many scholars have focused on signaling pathways and molecular mechanisms between PHB and breast cancer [43-46]. It is interesting that the mechanism of PHB, the same with other cancers, was not entirely consistent in breast cancer, more efforts are looking forward to elucidating it urgently. Our earlier study found an increased expression of PHB during the progression of normal breast tissue, hyperplasia tissue, atypical hyperplasia tissue, and breast cancer tissue [47], coincident with parts of research. Therefore, it has reason to believe PHB could serve as an essential factor in tumor development and a promising therapeutic target, including breast cancer [41, 43, 48]. However, the roles of PHB in cancer progression remain some controversial reports in the literature research. The present study aims to provide comprehensive and novel insights into the biological functions of PHB in the development, treatment, and prognosis of human pan-cancer, especially breast cancer, exploring the possible underlying molecular mechanisms associated with PHB in breast cancer.

## 2 MATERIALS AND METHODS

### 2.1 Bioinformatic Analysis of PHB Gene Expression and Functions

We analyzed the mRNA expression level of PHB in different tumor types based on the TGGA Database using an R package combined with standardized TPM expression data. Secondly, we performed a Protein-Protein Interaction (PPI) network in the STRING database (https://string-db.org/), a common online approach known to predict protein-protein interactions, to identify PHB-binding proteins and understand the possible underlying molecular mechanisms.

Furthermore, TIMER Database (cistrome.shinyapps.io/t) was used to perform correlation analysis between the expression levels of the *PHB* gene and tumor purity, as well as levels of tumor-infiltrating immune cells, including B cells, CD4+ T cells, CD8+ T cells, macrophages, neutrophils, and dendritic cells. Meanwhile, we downloaded the unified and standardized pan-cancer dataset: TCGA TARGET GTEx (PANCAN, N=19131, G=60499), extracted the expression level of PHB gene in each sample and extracted the gene expression profile of each tumor, and then calculated the StromalScore, ImmuneScore, and ESTIMATEScore of each tumor using R software package ESTIMATE algorithm. The correlation coefficient of genes and immune infiltration scores in each tumor was obtained by the Spearman test.

### 2.2 Survival Prognosis Analysis

PHB was evaluated for their prognostic value in breast cancer and other tumors using Kaplan–Meier Plotter (http://kmplot.com/analysis/), an online resource for survival analysis. Subsequently, the Kaplan–Meier method was used to analyze the overall survival (OS) of breast cancer patients, Kidney renal clear cell carcinoma, and so on based on the classification of patients into high and low groups according to their mRNA expression level. It was considered statistically significant using the criterion of log-rank *P* < 0.05.

### 2.3 Transient Transfection of MCF-7 and MTT Assay for Cell Viability

Human breast cancer cell MCF-7 was kindly provided by the Obstetrics and Gynecology Laboratory, First Affiliated Hospital of Xi’an Jiaotong University. It was cultured in Dulbecco's modified Eagle (DMEM) high glucose medium (HyClone, Logan, USA) containing 10% fetal bovine serum (Beyotime, Wuhan, China), 0.1% penicillin, and 0.1% streptomycin at 37℃ in 5% CO_2_ in the study. Enhance green fluorescence protein (pEGFP) (GenerayBiotech, Shanghai, China) was the vector of the recombinant plasmid. Transfection efficiency was confirmed by western blotting and real-time polymerase chain reaction (RT-PCR). Lipofectamine^TM^2000 reagent (Invitrogen, Carlsbad, USA) acted as the overexpressed group (OE group), transfection with an empty pEGFP vector was the negative control (NC group), only MCF-7 cells group was the mock group (MOCK group). Primers for PHB (Ref Seq: NM_003634) were designed using primer 3.0 and synthesized by Sangon Biotechnology Company (Shanghai, China). The primer sequence is shown as follows: Forward 5’-GGCTGAGCAACAGAAAAAGG-3’, and Reverse3’-TTGTAGTGGATGGACGGTCG-5’. The total ribose nucleic acid (RNA) of each group (OE, NC, and MOCK group) was extracted with Trizol reagent (Invitrogen, Waltham, USA) and quantified with Nanodrop2000 (Thermo Fisher Scientific, Waltham, USA). SYBR Green (Takara, Dalian, China) was utilized to mark real-time polymerase chain reaction (RT-PCR). Genomic deoxyribonucleic acid (DNA) was eliminated with a gDNA eraser kit reaction before conducting RT-PCR on a PCR Detection System (Thermo Fisher Scientific, Waltham, USA) with program firstly 95℃ for 30 seconds, secondly 40 cycles at 95℃ for 5 seconds and 55℃ for 30 seconds, thirdly 95℃ for 15 seconds and 60℃ for 30 seconds, at last 95℃ for 15 seconds. Fold change difference in messenger ribose nucleic acid (mRNA) expression of PHB was calculated using the 2^-△△Ct^ method which β-actin acted as an internal control.

Cell viability was detected with 3-(4, 5-dimethyl-2-thiazolyl)-2, 5-diphenyl-2-H-Tetrazoliumbromide (MTT). Cells were cultivated in triplicates of 96-well plates and transfected with 0.2 ug plasmid per well for the over-expressed group and negative control. After transfected for 24 hours, 48 hours, and 72 hours, respectively, the culture medium was gently removed and 20 ul 0.01 mol/L (5 mg/l) MTT was added to stain live cells for 4 hours at 37ºC in 5% CO_2_, the same with the MOCK group. Then, 100 ul dimethyl sulfoxide (DMSO) was added to each well and absorbance was measured by a full wavelength enzyme marker (TECAN, MäNeeded, Switzerland) at 490 nanometers. Cell growth inhibition rate was estimated with the equation 1- average OD of experiment group/OD of control.

### 2.4 Transwell Matrigel Invasion Assay

Firstly, matrigel (Sigma, San Francisco, USA) was previously diluted with DMEM medium (1:8) and 100ul suspension was added into the transwell chamber after 24 hours of transfection in different groups. After 48 hours of transfection, cells were collected, and the cell density was adjusted to 25×10^4^ cells/ml. Then, two hundred microliters of the cell solution were lightly dropped into each chamber and further incubated for 16 hours. Alcohol fixation and crystal violet staining were carried out finally. Cells penetrating through matrigel membranes of 10 fields were counted in each group randomly.

### 2.5 Cell Cycle and Apoptosis Analysis by Flow Cytometry

After transfection for 48 hours, cells were collected by trypsinization (no EDTA) and fixed with pre-cooled 75% ethanol overnight at 4℃, the same with the negative group and mock group. PropidiumIodide (PI) (Thermo Fisher Scientific, Waltham, USA) was added to stain the DNA for 30 minutes at room temperature in the dark. Flow Cytometer (Becton Dickinson, Franklin Lake, America) was used to detect DNA-PI fluorescence intensity at 530 and 488 nanometers, respectively, and Mod Fit LT was used to analyze the cell cycle.

We examined the apoptosis percentage of MCF-7 using flow cytometry with Annexin V-fluorescein isothiocyanate (FITC)/PI detection kit (KeyGENBioTECH, Nanjing, China). Cells were cultured in 6-well plates and transfected at 80% confluences. After 48 hours, cells were stained with Annexin V and analyzed by flow cytometry (cell Quest V.4.0) at 570 nanometers. The apoptosis rate was calculated with ModFit LT software.

### 2.6 Protein-protein Interactions Prediction and Checking for Tumor-Associated Proteins

STRING website, an online approach known to predict protein-protein interactions (PPI), showed PHB, P53, and E2F1 exhibited potential interactions. For western blotting, the total protein of each group was extracted from transfected cells after incubation for 72 hours with Radio-Immunoprecipitation Assay Buffer (RIPA) (HEART, Xi’an, China). All the lysates were added to a 5×loading buffer and stored at minus 80 ºC. Protein concentration was measured by BCA kit. Twenty micrograms of protein samples were isolated by Sodium Dodecyl Sulfate-Polyacrylamide Gel Electrophoresis (SDS-PAGE). The proteins were transferred onto polyvinylidene difluoride (PVDF) membrane and blocked in Tris-buffered saline and Tween-20 with 5% nonfat dry milk at room temperature for 2 hours. The membranes were incubated with primary antibodies (all primary antibodies: anti-prohibitin, anti-E2F1, anti-Bcl2, anti-βactin, and anti-erbB2 antibody from Abcam, Cambridge, UK) at 4 ℃ overnight. The membranes were washed with Tris-buffered saline (TBS) 3 times and incubated with HRP-linked secondary antibodies (Pierce Biotechnology, Rockford, USA) for 2 hours at room temperature. Meanwhile, the tumor-associated proteins, including P53, Bcl-2, E2F-1, and erbB-2 were detected. The signal was captured with Chemic Doc-It^TM^ UVP Imaging System Quantity One image processing software.

### 2.7 Statistical Analysis

SPSS Statistics (version 19.0 for Windows, IBM, Armonk, USA) was used for statistical analysis. Data are shown as mean ± standard deviation of 3 independent experiments. Comparisons among three groups were analyzed using one-way ANOVA and analysis of two groups was performed using unpaired two-tailed Student’s t-test. Graphs were generated by GraphPad Prism 5.0 software. A *p* value of < 0.05 was considered statistically significant.

## 3 RESULTS & DISCUSSION

### 3.1 Analysis of PHB Expression Level and Its Correlation with Tumor Immune Microenvironment

The pan-cancer analysis showed that *PHB* was over-expressed in most tumor types relative to normal tissues or paracological tissue based on the TCGA database, including in breast cancer (BRCA), as shown in Fig. (**1**) (*p* < 0.05). However, only three cancer types showed the opposite level of downregulation, including adrenal carcinoma (ACC), kidney renal clear cell carcinoma (KIRC), and acute myeloid leukemia (LAML). This result was consistent with most studies and our earlier research in breast cancer [47].

**Fig. (2):**
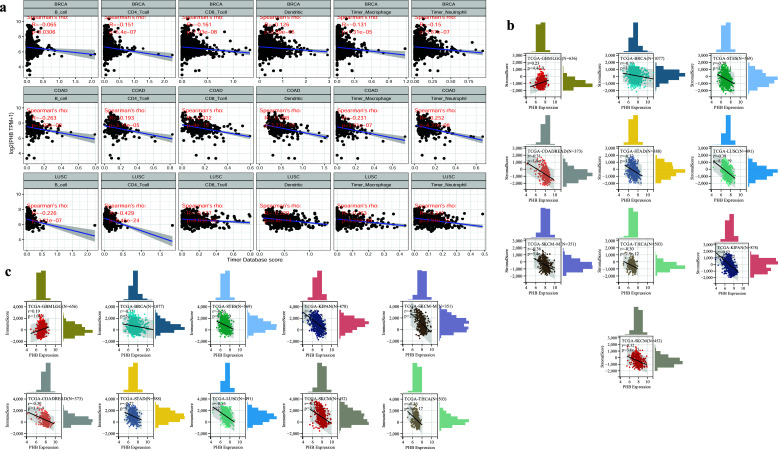
Correlation between expression of PHB and tumors immune infiltration. (**a**) Most tumor's immune infiltrating lymphocytes including B lymphocyte, CD4 T lymphocyte, CD8 T lymphocyte, Neutrophil, Macrophage, and Dendritic were all negatively correlated with expression of PHB. Breast cancer is one of the top 3 behind Chronic Obstructive Air Way Disease (COAD) and Lung Squamous Cell Carcinoma (LUSC) among 33 kinds of tumor samples. (**b**) Correlation between expression of PHB and Stromal score. (**c**) Correlation between expression of PHB and immune. The negative correlation between the expression of PHB and ImmuneScore (*R*= -0.116, *p* < 0.05) and StromalScore (*R*= -0.195, *p* < 0.05) of 33 kinds of tumor samples in the Timer database was obvious.

**Fig. (3):**
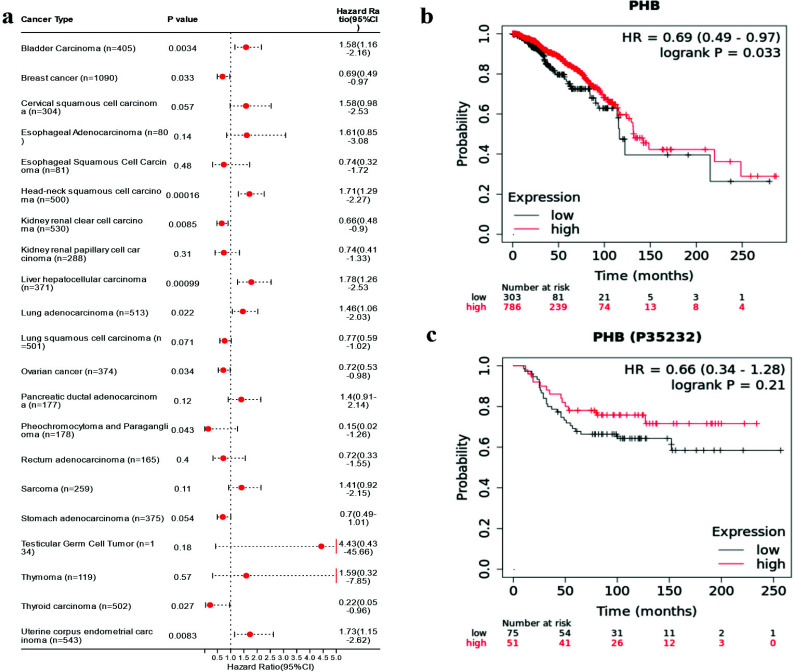
Survival prognosis analysis and Prognostic significance of PHB for pan-cancer. (**a**) Pan-cancer analysis revealed the prognosis correlation between the PHB gene and cancer patient OS. (**b**) The prognosis correlation between PHB gene and breast cancer. (**c**) The prognosis correlation between PHB protein and breast cancer.

Meanwhile, the negative correlation between tumor-infiltrating immune cells and *PHB* expression was evident (Fig. **2a**) in most tumor types using the Timer database, and breast cancer was located top 3 behind Chronic Obstructive Air Way Disease (COAD) and Lung Squamous cell Carcinoma (LUSC) as showed in Fig. (**2a**). Pan-cancer analysis including 33 kinds of tumors revealed the significantly negative correlations between PHB expression and ImmuneScore and StromalScore in most tumors (Fig. **2b** and **2c**), but among them, only the positive correlations in a few tumors including glioma (N = 656, R = 0.19, *P*= 1.0^e-6^ with ImmuneScore; R = 0.23, *P* = 4.4^e-9^ with StromalScore), uveal Melanoma (N = 79, R = 0.26, *P* = 0.02 with ImmuneScore; R = 0.24, P = 0.03 with StromalScore), acute Lymphoblastic Leukemia (N= 86, R = 0.23, *P* = 0.03 with ImmuneScore) (Fig. **3**).

### 3.2 Survival Prognosis Analysis and Prognostic Significance of PHB for Pan-cancer

We observed PHB gene was not only significantly over-expressed in most tumors compared with normal tissue, but also showed significant prognostic value by pan-cancer analysis. We noted that PHB mRNA should have a significant protective effect on the prognosis of breast cancer but with the lack of significant evidence that its protein form showed prognostic value on breast cancer. Therefore, we would further explore the biological effect of PHB on breast cancer cells through the overexpression plasmid recombinant plasmid pEGFP-PHB.

### 3.3 Efficiency of pEGFP-PHB Plasmid Transfection, Cell Proliferation, and Invasive Ability of PHB

**Fig. (4):**
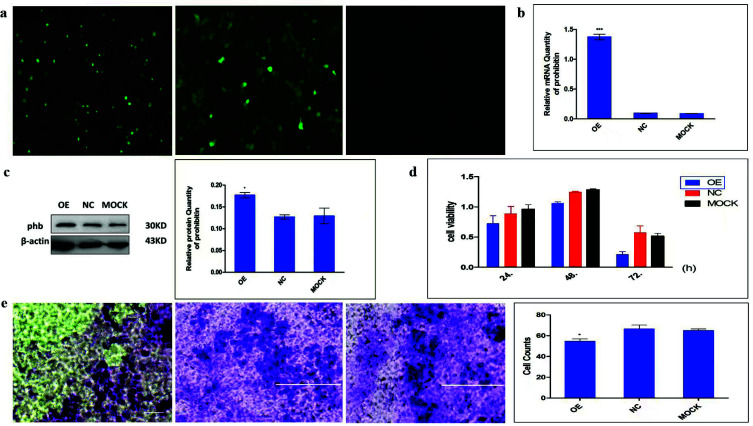
Transient Transfection of MCF-7, MTT Assay for Cell viability, and Invasion Assay of PHB. (**a**) Green fluorescence protein intensity among groups (OE, NC, and MOCK group ordinally). Cells were imaged with a fluorescence microscope (200×). Relative mRNA expression of PHB. GAPDH was used as an internal reference gene. (**b**) Representative western blot image of PHB and β-actin gene and densitometric analysis. (**c**) Cell viability was measured by MTT assay at 24, 48, or 72 hours following transfection of MCF-7 with pEGFP-PHB plasmid. (**d**) Representative transmembrane cell image of three groups after staining with crystal violet, and its statistical analysis of the total number of transmembrane cells in the three groups from 10 random fields. (**e**) the invasive ability of MCF-7 cells was measured by Transwell Ma trigel assay. *p < 0.001 or *p* < 0.05 *versus* NC control; **Abbreviations:** GAPDH=Glyceraldehyde 3-phosphate Dehydrogenase; OE=Overexpressed Group; NC=Negative Control Group.

Plasmid pEGFP-PHB was transiently transfected into MCF-7 breast cancer cells successfully and the transfection efficiency was measured by fluorescence microscopy, RT-PCR, and western blotting. As shown *in* Fig. (**4**), the fluorescence intensity of overexpressed-PHB cells and negative control was significantly higher than the MOCK control group, which indicates the model of over-expressed PHB was successfully constructed. Expressions of PHB at mRNA and protein levels were predominantly higher in the transfection group compared with the other two groups (Fig. **4b** and **4c**). Collectively, these results demonstrated that pEGFP-PHB was successfully transfected into the MCF-7 breast cancer cell, as well as increasing PHB expression.

To investigate the effect of PHB in tumor growth *in vitro*, an MTT assay was used to analyze cell viability at first. As shown in Fig. (**4**), all three groups showed a decreased proliferation in a time-dependent manner, and the overexpressed group showed more suppressive compared with the control cells (*p* < 0.05). The inhibition rates of proliferation were 20.98%, 14.93%, and 62.94% at 24, 48 and 72 hours, respectively in Fig. (**4d**). It was interesting that the inhibition rate was the most significant after 72 hours of transfection. Next, the effect of PHB on the invasive ability of MCF-7 cells was measured by transwell matrigel assay. As illustrated in Fig. (**4e**), the number of cells crossing the matrigel transwell membrane in the over-expressed group was 54.5 ± 7.4 compared with the negative control group (66 ± 12.6) and mock group (64 ± 7.4). These data suggested that PHB could decrease cell proliferation and invasive abilities *in vitro*.

### 3.4 PHB Influences Cell Cycle and Promotes Apoptosis Analysis

Cell proliferation and apoptosis in MCF-7 cells were tested by flow cytometry. The results showed that the cell percentage of the S phase in the overexpressed group, negative control, and mock group was 14.30 ± 6.97, 27.07 ± 4.44, and 26.54 ± 0.87, respectively. The cell percentages of the G2 phase were 34.76 ± 3.51, 21.57 ± 2.32, and 19.04 ± 2.83 in the over-expressed group, negative control, and mock group, respectively. The percentage of cells in the S phase was decreased, while those in the G2 phase were increased compared to the control groups in Fig. (**5a**-**5d**). No significant difference was observed in the G1 phase among the three groups (*p* > 0.05). The results indicated that PHB changed the cell cycle distribution, arrested DNA synthesis in the S phase, and induced cell cycle S/G2 arrest. Thereby inhibiting cell proliferation in ER-positive breast cancer cells MCF-7. Moreover, cellular apoptosis was higher in the over-expressed group *vs.* controls both in the early and late stages of apoptosis in Fig. (**5e**-**5f**). The apoptosis rate in the over-expressed group was 33.67 ± 8.68% compared with the negative control group (12.13 ± 3.76%) and the mock group (6.99 ± 2.33%) (*p* < 0.05). Collectively, these data further suggested that PHB inhibited breast cancer cell proliferation and promoted cell apoptosis.

### 3.5 PHB Influences Expression of Multiple Tumors Associated Proteins

**Fig. (6):**
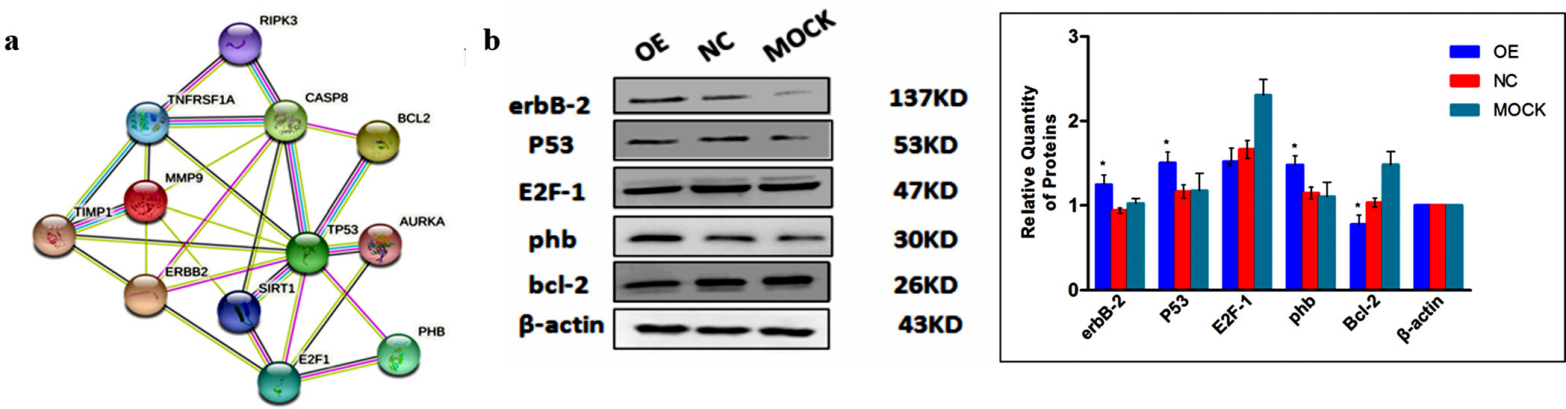
Effect of PHB on tumor-associated proteins. (**a**) PHB-binding proteins and PPI network. (**b**) Representative western blot image. KD = kilodalton; OE = over expressed group; NC= negative control, β-actin was used as a loading control. (**c**) Statistic analysis of tumor correlative protein levels. OE = overexpressed group; NC = negative control group, *p < 0.05 *vs.* negative control.

We explored related molecular mechanisms of PHB regulating cell growth and apoptosis as the results showed above that PHB inhibits the MCF-7 cell growth. STRING website, an online approach known to predict protein-protein interactions (PPI), showed PHB, P53, and E2F1 exhibited the potential interactions (Fig. **6a**). Several critical indicators, including erbB-2, tumor gene P53, transcription factor E2F-1, and cell cycle protein Bcl-2, were measured by western blotting. As shown in Fig. (**6b**), erbB-2, which is closely related to breast cancer diagnosis and treatment, and P53, a tumor suppressor protein, were increased when PHB overexpressed. In contrast, PHB decreased the expression level of cell cycle protein Bcl-2, an anti-apoptotic protein. However, the transcription factor E2F-1 remained unchanged. These results suggested that PHB modulates tumor cell growth and apoptosis by regulating the expression of proteins that are associated with tumor development (Fig. **6c**).

## CONCLUSION

The morbidity and mortality rates of breast carcinoma rank high for women all over the world [26, 49]. It is not linked only to its unique biological characteristics, but also to close living environment [10, 50]. It is exciting that current scientific screening techniques and molecular diagnosis research have greatly reduced the mortality rate of early breast cancer [51, 52]. In Combination with the heterogeneity and specificity of breast cancer, non-responsiveness or resistance to hormone-related drugs, and complexity of molecular mechanisms, comprehensive consideration in the process of the screening, diagnosis, and treatment of breast cancer is needed, such as age, hormone level, drug resistance, races, and economic level [14, 28, 49, 53-56]. More, current treatments for breast cancer are expected to extend 5-year survival and improve life quality based on the control tumor development [6]. Biomarkers and precision therapy the promising prospects for breast cancer patients [57, 58].

*PHB* was one of the potential genes in tumorigenesis for breast cancer [59]. Many studies have shown that it is highly expressed in breast cancer, the same with our early study [47]. It is a highly homologous protein in a variety of cancer cells linked to cell proliferation, apoptosis [30], single transduction, transcription, energy metabolism, and immune regulation [58]. What’s more, PHB was reported to express differently under the inflammatory stimulation of cancers and diseases [41, 59-66]. PHB was shown to be linked to kinds of signal paths such as Ras-Raf-MEK-ERK [23], P53 signal pathway [38], and Rb-E2Fs signal pathway [64], and so on [65, 66]. When it comes to PHB in breast cancer, mRNA encoded by 3 '-UTR of PHB was directly microinjected into the nucleus of MCF-7 breast cancer, and the cell cycle was blocked in the G1/S phase [67]. PHB at a physiological dose can not only interact with P53 and enhance its ability to bind to DNA promoters, inhibiting cell proliferation subsequent, but its overexpression also increases the transcriptional activity regulated by P53 and inhibits the transcriptional activity regulated by E2F1 [38]. PHB combines with Rb and E2Fs in the nucleus to form a complex triplet, which prevents the binding of E2Fs to the DNA promoter, thus inhibiting the transcriptional activity of E2F and preventing the cell from entering the S phase from G1 [64]. All these indicated that breast cancer could be linked with PHB through multipaths [68]. The correlation of PHB expression and 33 kinds of tumor samples was negative, with the top 3 based on bioinformatic analysis in this study. PHB was expected as a biomarker of the diagnosis, therapy, and prognosis of diseases, especially for breast cancer [5, 45, 54, 69, 70].

To further explore the function of PHB in breast cancer tumorigenesis following a previous study, we first explored bioinformation roles based on the Cancer Genome Atlas (TGGA) Database and conducted biological functions *in vitro*. Significant upregulation of PHB could be found in different tumors, compared to normal tissues, including breast cancer, lung adenocarcinoma, and non-small cell lung cancer. High PHB mRNA levels were associated with a favorable prognosis in breast cancer patients, but in fact, the potential effect of PHB on breast cancer needed further study. Notably, our study found that PHB showed a favorable or unfavorable prognostic effect in different tumors by pan-cancer analysis. Therefore, the distinct roles of PHB in breast cancer remain to be elucidated by a series of experiments to determine whether PHB acts as a tumor suppressor molecule in breast cancer.

Based on pan-cancer analysis using bioinformatics methods and our previous study [47], we explored the biological functions of PHB in MCF-7 human breast cancer cells by transfection with pEGFP-PHB plasmid. Our data indicate that higher expression of PHB inhibited cell proliferation, arrested cell cycle in the S/G2 phase, decreased cell viability and invasive ability of MCF-7, and accelerated cell apoptosis *in vitro*. Moreover, these changes were likely related to the expression of tumor-associated proteins such as P53, erbB-2, Bcl-2, *etc.* Previous studies have shown that PHB could individually or jointly act with oncogene P53 [38], positive transcription factor E2F-1, and Rb family members to inhibit cell proliferation. Meanwhile, regulatory factors such as BrgI, Brm, HDAC1, and HP1 are also involved in proliferation. Further studies discovered that the Ras-Raf-MEK-ERK signal pathway might affect proliferation [23]. In this study, prohibitin changed cell cycle distribution and arrested cells in the S/G2 phase. Moreover, cell viability was reduced compared with control cells. All these results demonstrate that PHB inhibited breast cancer cell DNA replication and affected cell cycle distribution, thereby restraining cell viability and cell proliferation. The data involved in cellular apoptosis showed that the percentage of cells was increased in the over-expressing prohibitin, no matter the early stages and the later stages of apoptosis. As some studies indicated that P53 could not only inhibit cell proliferation but also mediate cell apoptosis [30, 38], our study also supported this possibility, as we observed an increased expression of P53 in cells with overexpressed PHB. As apoptosis tightly depends on the balance of pro-apoptosis proteins (*e.g.,* Bax, Bak, and Noxa proteins) and the opposite anti-apoptosis proteins (*e.g.,* Bcl-2, Bcl-xl, and Bcl-w), any change in the expression of these proteins could induce or inhibit apoptosis [30]. In the present study, the reduction of Bcl-2 was more likely responsible for the induction of apoptosis by PHB. Consistently, cell invasive ability was decreased which may be associated with an increase in apoptosis of the over-expressing group. A human epidermal growth factor erbB-2 [17], also known as Her-2, triggers cell invasive ability, and a high level of Her-2 could promote cancer cell growth, invasive ability, and resistance to chemotherapy drugs in ER-positive cells. We found that Her-2 was increased, and the invasive ability was reduced which indicates PHB could regulate Her-2 through unclear downstream mechanisms, further studies are required to explore this possibility.

Finally, Tumor Microenvironment (TME) was essential in the development and progression of cancer [50, 57]. Many recent studies have demonstrated that tumor-infiltrating immunocytes (TICs) could harbor either tumor-promoting or tumor-suppressing activities due to the regulation of tumor progression, invasion, metastasis, and lymph node metastasis. Our study showed that the expression of PHB significantly correlated with the infiltration of six immune cell types, including B cells, CD4+T cells, and CD8+ T cells, suggesting that PHB could also indicate the immune status besides the disease prognosis.

Under the guidance of bioinformatic analysis for pan-cancer, we have successfully constructed the model of higher PHB breast cancer cell with transient transfection, and explored the biological functions that inhibited cell proliferation, arrested cell cycle in the S/G2 phase, decreased cell viability, and invasive ability of MCF-7, and accelerated cell apoptosis *in vitro,* this study indicate PHB regulates breast cancer cell survival and predicts biomarker function. However, there are many drawbacks in the present study. In addition, to improve this experiment, we intend to further study the expression level of PHB in the tissue and serum samples from breast cancer patients or use gene sequencing technology to validate the biomarker role and clinical value of PHB.

## Figures and Tables

**Fig. (1):**
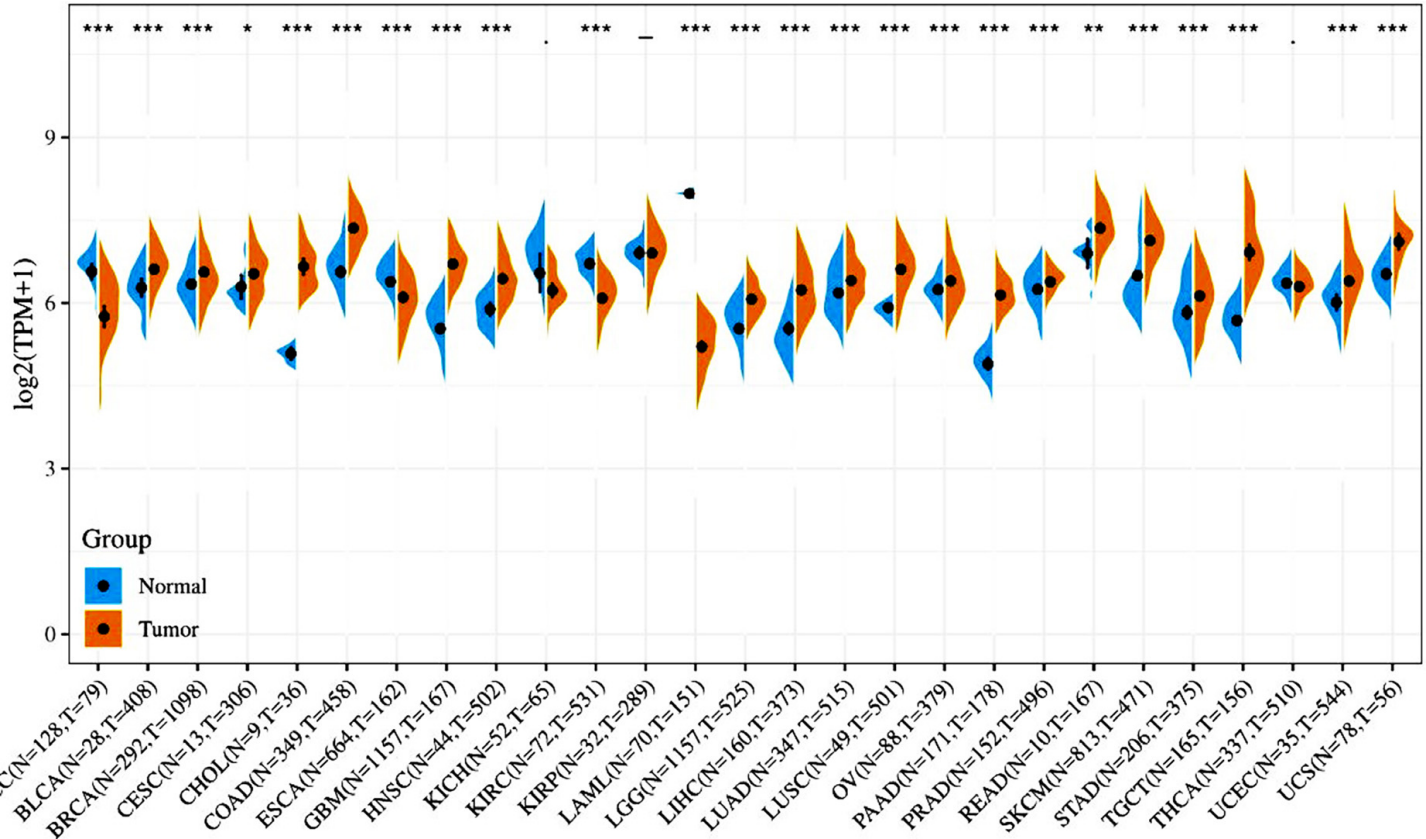
Expression of PHB in a multitude of tumors. The differences in expression levels of PHB mRNA in 27 kinds of tumors and normal tissues from TCGA and GTEx database.

**Fig. (5):**
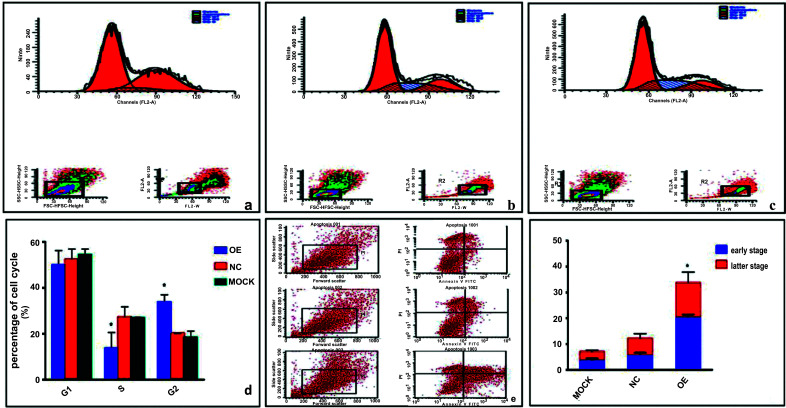
Effect of PHB on proliferation and apoptosis in MCF-7 cells. (**a-d**) The distribution of cell cycle among the three groups by flow cytometry. Cells were collected and washed with phosphate-buffered saline fixed with alcohol, and stained with DNA with propidiumiodide. (**e** and **f**) Effect of PHB on apoptosis. After transfection with the plasmid for 48 h, cells were collected and stained with annexin. Propidium Iodide and fluorescein isothiocyanate (FITC) were used to stain phosphatidylserine in the early apoptosis stage and DNA in the later apoptosis, respectively. Analysis of variance was used for comparison. OE = overexpressed group; NC = negative control group, *p < 0.05 *vs.* controls.

## Data Availability

We declare that all data and materials are valid and available.

## LIST OF ABBREVIATIONS

ACC: Adrenal Carcinoma
BRCA: Breast Cancer
BRCA1: Breast Cancer Susceptibility Gene 1
COAD: Chronic Obstructive Air Way Disease
DMEM: Dulbecco's Modified Eagle
DNA: Deoxyribonucleic Acid
KIRC: Kidney Renal Clear Cell Carcinoma
LAML: Acute Myeloid Leukemia
NC group: Negative Control
OE group: Overexpressed Group
OS: Overall Survival
PHB/phb: Prohibitin
PI: PropidiumIodide
PPI: Protein-protein Interaction
PVDF: Polyvinylidene Difluoride
RIPA: Radio-Immunoprecipitation Assay Buffer
RNA: Ribose Nucleic Acid
RT-PCR: Real-time Polymerase Chain Reaction
TBS: Tris-buffered Saline
TCGA: The Cancer Genome Atlas
TICs: Tumor-infiltrating Immunocytes
TME: Tumor Microenvironment
